# Supplementary material for: The influence of 4-week eccentric Nordic hamstring exercise training on postural balance and muscle strength: A randomized controlled trial
Source: PLoS One. 2025 Jun 17;20(6):e0315459. doi: 10.1371/journal.pone.0315459 (PMC12173193; doi:10.1371/journal.pone.0315459)
Supplement: S1 File — (DOC) [file pone.0315459.s001.doc]

# Wniosek o wydanie opinii o badaniu nie sponsorowanym

Tytuł badania (w języku polskim): Ocena wpływu 4-tygodniowego treningu ekscentrycznego typu Nordic Hamstring Exercise na siłę i równowagę grupy mięśni kulszowo-goleniowych.

INFORMACJE O GŁÓWNYM BADACZU

Imię i nazwisko głównego badacza: Magdalena Podczarska-Głowacka

Specjalizacja: fizjoterapia

Tytuł naukowy: doktor nauk o kulturze fizycznej

Kierownik tematu badawczego: dr Magdalena Podczarska-Głowacka

INFORMACJE O BADANIU

Pełna nazwa miejsca prowadzenia badania: Akademia Wychowania Fizycznego i Sportu w Gdańsku

Do zespołu badaczy należą (imię, nazwisko, specjalizacja, tytuł naukowy):

1. Magdalena Podczarska-Głowacka, fizjoterapia, doktor
2. Katarzyna Krasowska, fizjoterapia, doktor
3. Agata Kalkowska, fizjoterapia, magister
4. Zuzanna Trapik, fizjoterapia, magister

RODZAJ BADANIA (np. praca doktorska, grant): projekt badawczy

INFORMACJA O BADANIU:

Celem badania jest ocena wpływu 4 tygodniowego treningu ekscentrycznego Nordic Hamstring Exercise na zmiany w obrębie grupy mięśni kulszowo-goleniowych, a także analiza parametrów takich jak maksymalna siła mięśniowa zginaczy stawu kolanowego, wskaźnik rozwoju siły RFD (rate of force development) oraz równowaga w statyce, dynamice dla nogi dominującej.

Na podstawie analizy piśmiennictwa postawiono następujące hipotezy:

1. Trening 4-tygodniowy Nordic Hamstring Exercise wpłynie znacząco na siłę grupy tylnej mięśni uda, co przełoży się zwiększeniem siły mięśniowej po przebytym treningu.
2. 4-tygodniowy protokół ekscentryczny Nordic Hamstring Exercise wpłynie na polepszenie równowagi w statyce i dynamice.
3. Po zakończonym treningu Nordid Hamstring Exercise nastąpi istotny wzrost siły w jak najkrótszym czasie.

Procedura:

W badaniu wezmą udział studenci Akademii Wychowania Fizycznego i Sportu w Gdańsku w wieku od 19-26 lat (n=30) uprawiający amatorsko piłkę nożną. Badani podzieleni zostaną losowo na dwie grupy badawcze. Pierwsza grupa badawcza prowadzona będzie 4-tygodniowym treningiem Nordic Hamstring Exercise, druga grupa badawcza będzie grupą kontrolną wykonującą tradycyjne ćwiczenia. Przed procedurą eksperymentalną podpiszą świadomą, dobrowolną zgodę oraz zostaną poinstruowani o badaniu.

I Faza badania – Faza Familaryzacji:

Tydzień przed rozpoczęciem badania eksperymentalnego uczestnicy zostaną zaproszeni do Laboratorium Wysiłku Fizycznego w AWFiS w Gdańsku celem zapoznania się z procedurą ćwiczeń Nordic Hamstring, z aparaturą na której prowadzone będzie badanie oraz z osobami, które przeprowadzą to badanie.

II Faza badania – Faza Właściwa:

Procedura badań: Trening Nordic Hamstring Exercise obejmuje okres 4 tygodni, w których ochotnik ćwiczy 3 razy w tygodniu po 3 serie, każda seria składa się z 3 powtórzeń NHE. Przed rozpoczęciem ćwiczeń ekscentrycznych poprowadzi się rozgrzewkę przez okres 15 minut. Natomiast po każdej serii nastąpi 2 minutowa przerwa pasywna, w której osoba badana odpocznie. W pierwszym tygodniu badany będzie ćwiczył pod okiem prowadzącego, w kolejnych trzech tygodniach badany ćwiczy w warunkach domowych po wcześniejszym instruktarzu.

Ćwiczenia Nordic Hamstring Exercise polegają na kontrolowanym opadzie wyprostowanego tułowia z pozycji pionowej w klęku do przodu w stronę podłoża (równolegle do podłoża) i powrocie do pozycji wyjściowej. Stawy skokowe podczas serii ćwiczeń ustabilizowane są przez chwyt drugiej osoby. Jeśli badany będzie miała problem z utrzymaniem tułowia w warunkach kontrolowanej pracy ekscentrycznej wówczas kończyny górne powinny zabezpieczać i amortyzować dotknięcie podłogi, po czym nastąpi odbicie z dłoni do pozycji wyjściowej. Po każdej sesji następuje 2 minutowa przerwa pasywna.

Przy użyciu Systemu Biodex dokonana zostanie ocena maksymalnej siły mięśniowej kończyny dolnej dla prostowników stawu kolanowego oraz wskaźnik rozwoju siły RFD (rate of force development). Wskaźnik RFD charakteryzuje zdolność organizmu do rozwijania maksymalnej siły (mocy) w możliwym krótkim czasie. Pomiary dokonane zostaną dwukrotnie, przed jak i po zastosowanej procedurze treningowej.

Do pomiaru równowagi statycznej i dynamicznej wykorzystany zostanie Biodex Balance System (BBS). Pozwalający ocenić przyśrodkowo-boczny wskaźnik stabilności (MLSI), przednio-tylny wskaźnik stabilności (APSI) i ogólny wskaźnik stabilności. Procedura pomiarowa będzie przeprowadzona przed i po procedurze badawczej.

Kryteria doboru do badania eksperymentalnego:

- osoby zdrowe w wieku 19-26 lat,

- bez kontuzji,

- sportowcy, osoby amatorsko uprawiające sport.

Kryteria wykluczenia z badania eksperymentalnego:

- w okresie największych obciążeń treningowych związanych z dyscypliną,

- zmiany przeciążeniowe w obrębie stawów: kolanowych, biodrowych i w odcinku lędźwiowo-krzyżowym,

- dolegliwości bólowe,

- stany zmęczeniowe mięśni, przetrenowanie,

- w sportach siłowych na kilka tygodni przed startem,

- stany zapalne elementów kostno-stawowo-więzadłowych kończyn dolnych,

- przebyte kontuzje, zabiegi chirurgiczne w czasie 6 miesięcy przed interwencją.

Przewidywany termin rozpoczęcia badania: styczeń 2024

Przewidywany termin zakończenia badania: marzec 2024

**Dokumenty dołączone do wniosku (prosimy podać dokładne wersje, daty):**

Informacja o badaniu;

Życiorys głównego badacza;

Informacja dla Pacjenta i Formularz Świadomej Zgody;

Wzór zgody Pacjenta na przetwarzanie danych osobowych;

Zgoda Rektora AWFiS w Gdańsku na przeprowadzenie badania;

**Application for an opinion on a non-sponsored study**

Title of the study (in Polish): Assessment of the impact of 4-week Nordic Hamstring Exercise eccentric training on the strength and balance of the hamstring muscle group.

INFORMATION ABOUT THE PRINCIPAL INVESTIGATOR

Name and surname of the main researcher: Magdalena Podczarska-Głowacka

Specialization: physiotherapy

Scientific title: PhD in physical culture

Research topic leader: Dr. Magdalena Podczarska-Głowacka

INFORMATION ABOUT THE TEST

Full name of the place where the study was conducted: Gdansk University of Physical Education and Sport

The team of researchers includes (name, surname, specialization, academic title):

1. Magdalena Podczarska-Głowacka, physiotherapy, Ph.D

2. Katarzyna Krasowska, physiotherapy, Ph.D

3. Agata Kalkowska, physiotherapy, master's degree

4. Zuzanna Trapik, physiotherapy, master's degree

TYPE OF RESEARCH (e.g. doctoral thesis, grant): research project

INFORMATION ABOUT THE TEST:

The aim of the study is to assess the impact of 4 weeks of eccentric Nordic Hamstring Exercise training on changes in the hamstring muscle group, as well as to analyze parameters such as maximum muscle strength of the knee flexors, RFD (rate of force development) and static balance, dynamics for the dominant leg.

Based on the literature analysis, the following hypotheses were formulated:

1. The 4-week Nordic Hamstring Exercise training will significantly improve the strength of the rear group of thigh muscles, which will translate into increased muscle strength after training.

2. The 4-week Nordic Hamstring Exercise eccentric protocol will improve static and dynamic balance.

3. After completing the Nordid Hamstring Exercise training, you will experience a significant increase in strength in the shortest possible time.

Procedure:

The study will involve students of the Academy of Physical Education and Sport in Gdańsk, aged 19-26 (n=30) who practice amateur football. The subjects will be randomly divided into two research groups. The first research group will undergo a 4-week Nordic Hamstring Exercise training, the second research group will be a control group performing traditional exercises. Before the experimental procedure, they will sign an informed, voluntary consent and will be instructed about the study.

Phase I of the study – Familarization Phase:

A week before the start of the experimental study, participants will be invited to the Physical Exercise Laboratory at AWFiS in Gdańsk to familiarize themselves with the Nordic Hamstring exercise procedure, with the equipment on which the study will be conducted and with the people who will conduct the study.

Phase II of the study - Proper Phase:

Research procedure: Nordic Hamstring Exercise training covers a period of 4 weeks, during which the volunteer exercises 3 times a week for 3 sets, each set consisting of 3 repetitions of NHE. Before starting eccentric exercises, you will warm up for 15 minutes. After each series, there will be a 2-minute passive break, during which the subject will rest. In the first week, the subject will exercise under the supervision of the instructor, in the next three weeks the subject will exercise at home after prior instruction.

Nordic Hamstring Exercise involves a controlled descent of the upright torso from a vertical kneeling position forward towards the ground (parallel to the ground) and returning to the starting position. The ankle joints are stabilized by the other person's grip during a series of exercises. If the subject has difficulty maintaining the torso in conditions of controlled eccentric work, the upper limbs should protect and absorb the impact of the floor, after which the hand will bounce back to the starting position. After each session there is a 2-minute passive break.

Using the Biodex System, the maximum muscle strength of the lower limb for the knee joint extensors and the RFD (rate of force development) index will be assessed. The RFD index characterizes the body's ability to develop maximum strength (power) in the shortest possible time. Measurements will be taken twice, before and after the training procedure.

The Biodex Balance System (BBS) will be used to measure static and dynamic balance. Evaluating the medial-lateral stability index (MLSI), anteroposterior stability index (APSI) and overall stability index. The measurement procedure will be carried out before and after the research procedure.

Selection criteria for the experimental study:

- healthy people aged 19-26,

- no injuries,

- athletes, people practicing sports as amateurs.

Exclusion criteria from an experimental study:

- during the period of greatest training load related to the discipline,

- overload changes in the joints: knee, hip and lumbar-sacral,

- pain,

- muscle fatigue, overtraining,

- in strength sports a few weeks before the start,

- inflammation of the osteoarticular and ligamentous elements of the lower limbs,

- previous injuries, surgical procedures within 6 months before the intervention.
